# Comparison of preoperative angle kappa measurements in the eyes of cataract patients obtained from Pentacam Scheimpflug system, optical low-coherence reflectometry, and ray-tracing aberrometry

**DOI:** 10.1186/s12886-021-02116-w

**Published:** 2022-04-02

**Authors:** Miaomiao Qin, Yurong Yuan, Ying Wang, Pengfei Li, Wei Chen, Yong Wang, Mei Yang, Jian Wu, Min Ji, Jiawei Luo, Jiamin Tang, Xiaojuan Chen, Yemeng Huang, Huaijin Guan

**Affiliations:** Eye Institute, Affiliated Hospital of Nantong University, 20 Xisi Road, Nantong, 226001 Jiangsu China

**Keywords:** Angle kappa, Cataract, Pentacam, Lenstar, iTrace

## Abstract

**Background:**

Angle kappa plays a vital role in the implantation of multifocal intraocular lens (MIOL). Large angle kappa is related to a higher risk of postoperative photic phenomena. This study aims to compare preoperative angle kappa in the eyes of cataract patients obtained from the Pentacam Scheimpflug system (Pentacam), optical low-coherence reflectometry (Lenstar), and ray-tracing aberrometry (iTrace).

**Methods:**

One hundred thirteen eyes of 113 patients with cataracts were included. Each eye was examined 3 times using all devices to obtain angle kappa and pupil diameter. When considering dependent eyes for one individual, angle kappa in both right eyes and left eyes should be analysed separately. The repeatability and reproducibility were evaluated using the within-subject standard deviation (Sw), repeatability (2.77 Sw), and intraclass correlation coefficient (ICC). The difference, correlation, and agreement between devices were evaluated by paired *t*-tests, Pearson tests, and Bland-Altman analysis, respectively.

**Results:**

Intraoperator repeatability and interoperator and intersession reproducibility of angle kappa showed an Sw of less than 0.05 mm, a 2.77 Sw of 0.14 mm or less, and an ICC of more than 0.96. Angle kappa was not significantly different between Pentacam and Lenstar (*P* > 0.05), while angle kappa was significantly different between Pentacam and iTrace and between Lenstar and iTrace (*P* < 0.05). There was a strong correlation between Pentacam and Lenstar for angle kappa (*r* =0.907 to 0.918) and a weak or moderate correlation between Pentacam and iTrace and between Lenstar and iTrace (*r* =0.292 to 0.618). There were narrow 95% limits of agreement (LoA) between Pentacam and Lenstar for angle kappa and wide 95% LoA between Pentacam and iTrace and between Lenstar and iTrace. No significant differences in pupil diameter were found between Pentacam and Lenstar in either eye (*P* > 0.05). Positive angle kappa (nasal light reflex) was found in most cataract patients (79.25% to 84.91%) through 3 different devices in both eyes.

**Conclusions:**

The 3 devices provided high intraoperator repeatability and interoperator and intersession reproducibility for angle kappa measurements. The measurement of preoperative angle kappa in the eyes of patients with cataracts by Pentacam and Lenstar has good agreement.

## Background

Angle kappa represents the angle between the visual axis and the pupillary axis [1]. The visual axis connects the fovea with the fixation point; this line passes the nodal, and the pupillary axis is the line passing through the center of the pupil perpendicular to the cornea. According to the light reflex located in the pupillary center, angle kappa can be classified as positive (nasal) or negative (temporal). Additionally, angle kappa can be classified as horizontal or vertical on the basis of X and Y Cartesian values of angle kappa. A positive angle kappa of 5.0° on average is generally found in the normal human eye [2].

Angle kappa is a crucial examination for some surgical decisions in ophthalmology. In keratorefractive surgery with a large angle kappa, there is a greater chance of the decentration of ablation zones, and it may lead to negative visual effects such as irregular astigmatism and undercorrection [3, 4]. Similarly, during intraocular refractive surgery implantation of the intraocular lens (IOL), especially the multifocal intraocular lens (MIOL), a large angle kappa can increase the risk for photic phenomena including halos, glare, and dysphotopsia [5, 6]. Therefore, angle kappa is clinically significant to consider in the preoperative assessment of patients.

Many devices have been commercially released for measuring angle kappa. The exact angle kappa is commonly measured using a synoptophore or major amblyoscope which has not become commercially available [1]. With the improvements of higher precision in biometers, newer instruments measuring angle kappa are applied in clinical practice. Pentacam has been increasingly popular in measuring angle kappa for the past few years [7]. In addition, some reports have shown that Lenstar and iTrace are also commonly used to estimate angle kappa [8, 9]. However, no published data have reported a comparison in the angle kappa among three devices. The aim of this study was to compare preoperative angle kappa in the eyes of cataract patients obtained from those instruments and to provide a reference for the selection and judgement of surgeons.

## Methods

### Subjects

This retrospective study enrolled 113 eyes of 113 patients with cataracts who attended the Department of Ophthalmology, Affiliated Hospital of Nantong University, between October 2018 and December 2019 for cataract surgery. The study was approved by the ethics committee of the Affiliated Hospital of Nantong University and complied with the tenets of the Declaration of Helsinki. All patients were willing to volunteer for the research and signed a written informed consent form.

All patients underwent a comprehensive ophthalmologic examination, including uncorrected and best-corrected visual acuity, manifest refraction, pupil diameter, intraocular pressure, slit-lamp anterior segment evaluation, and fundus examination with the pupil dilated. Inclusion criteria included age-related cataract patients with lens opacity grading from C1N1P1 to C3N3P3 according to LOCS III, and eyes with preoperative uncorrected distance visual acuity (UDVA, recorded in logMAR units) less than 0.7 (better visual acuity). Exclusion criteria were any corneal opacities, poor fixation, strabismus, dry eye (dry eye symptoms or break-up time shorter than 5 seconds), keratoconus, a history of ocular surgery for refractive error and trauma, use of contact lenses, and other ocular pathology or neurological lesions that might affect vision.

### Angle Kappa measurements

Angle kappa data were recorded by three different instruments (Pentacam, Lenstar and iTrace). First, the angle kappa was displayed through X-Y Cartesian coordinates. According to X-Y Cartesian coordinates, the angle kappa distribution can be classified into the following 8 positions: superior nasal, inferior nasal, superior temporal, inferior temporal, nasal, temporal, superior and inferior. Second, the values of X and Y with different head positions changed, but the chord length of X and Y did not change with different head positions. The size of the angle kappa was the chord length of X and Y.

### Scheimpflug system measurements

**Fig. 1:**
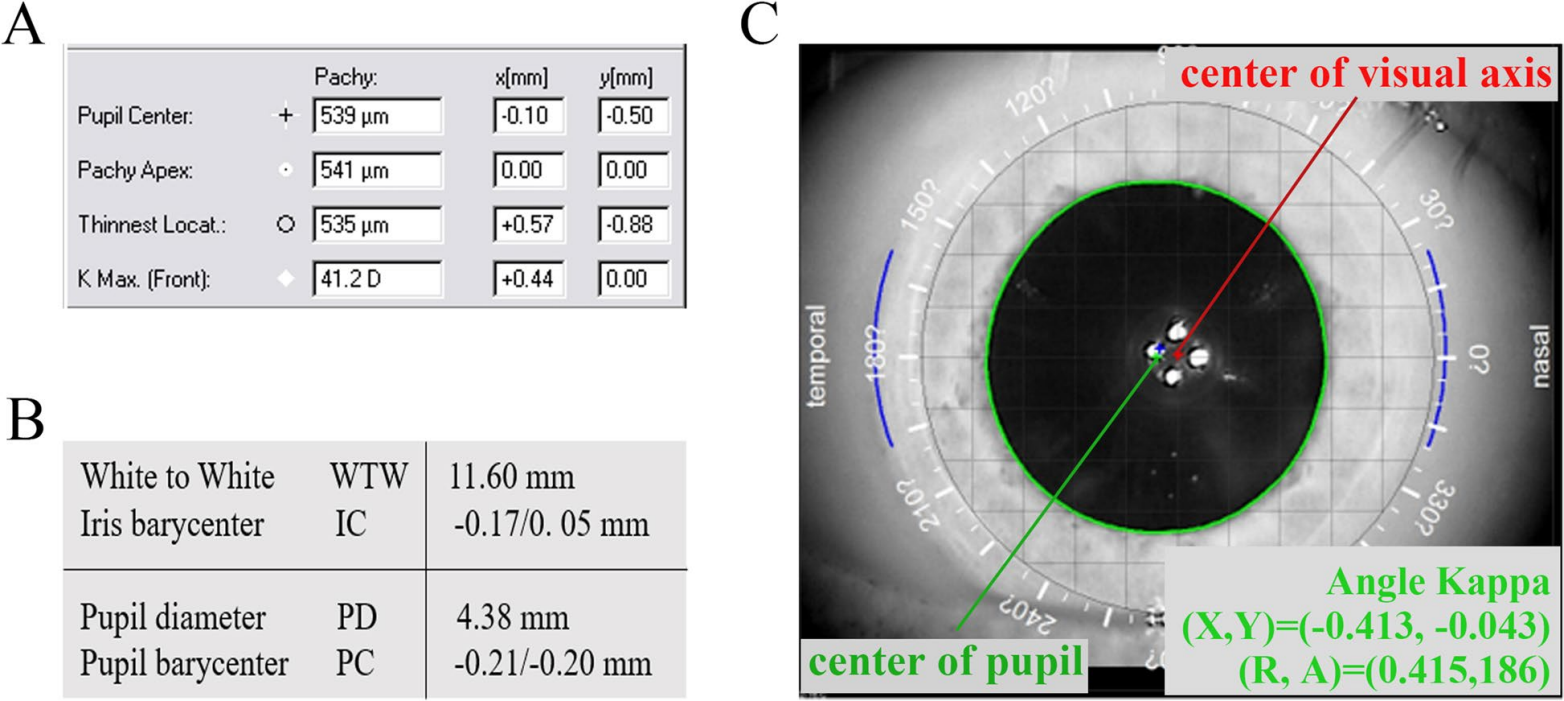
Angle kappa displayed through X-Y Cartesian coordinates measured by Pentacam (A), Lenstar (B) and iTrace (C). Aberrometer image (C) shows the center of the visual axis (red cross [C], representing the center of the 4 white reflection points) and pupillary center (green cross [C], representing the center of the green circle).

The Pentacam system (70700; Oculus, Wetzlar, Germany) is a Scheimpflug-based instrument that obtains a three-dimensional model of the anterior segment of the eye that indicates an image from the anterior corneal surface to the posterior lens surface. The device captures up to 25 slit-images of the anterior segment of the eye by a 360-degree rotating Scheimpflug camera, collecting 25000 true elevation data points (respecting 500 true elevation points per slit image) within 2 seconds. The patients placed their chins on a chin rest, forehead against a forehead strap and looked at the fixation target according to the manufacturer’s instructions. The operator visualizes a real-time image of the patient’s eye on a computer screen, with the machine marking the pupil edge and center and the corneal apex. Arrows are shown on the screen, which guide the operator’s alignment of the device in the horizontal, vertical, and translatory axes. Angle kappa was measured by the coordinates (X, Y) between the center of the corneal vertex and the pupil (Figure 1, A), and the automatic release mode was used to reduce operator-dependent variables.

### Optical low-coherence reflectometer measurements

The noncontact ocular biometry Lenstar (LS900; Haag-Streit, Koniz, Switzerland) calculates ocular distances by the effect of time domain interferometric or coherent superposition of light waves. It is based on optical low-coherence reflectometry using a 20~30 nm broadband light source with an 820 nm center wavelength. The patients seated with his or her chin on a chin rest and pressed their forehead against the forehead strap. The Lenstar was focused and aligned using eye images on the computer monitor while the patients were asked to fixate straight ahead on an internal fixation light. The mean value of those measurements was automatically calculated by the instrument software. The eccentricity of the visual optical line was assessed according to the distance of the visual axis and the pupil center. Angle kappa was calculated using the X and Y coordinates of the pupil barycenter (pupil dx, pupil dy) (Figure 1, B). Pupil dx indicates the x coordinate of the pupil center relative to the corneal apex, and pupil dy indicates the y coordinate of the pupil center relative to the corneal apex. The size of the angle kappa was calculated using the formula r = (X^2^ +Y^2^) ^1/2^.

### Ray-tracing aberrometry

The iTrace aberrometer analyser (iTrace; Tracey Technologies, Houston, USA) combines ray-tracing aberrometry and corneal topography. The ray-tracing principle which sequentially projects 256 near-infrared laser beams with a 785 nm center wavelength into the eye in a specific scanning pattern is used for the aberrometer. Placido-based corneal topographer is captured by topographies. The subjects placed their chin on the chin rest, pressed their forehead against the strap and were asked to fixate on the red light. An iris image with an infrared camera was captured automatically by the aberrometer to display angle kappa. Angle kappa was displayed by the coordinates (X, Y) between the center of the visual axis and the pupil, and the magnitude of angle kappa was assessed between the chord length of X and Y (Figure 1, C).

### Measurement protocol

The whole protocol can be divided into two sessions. In the first session, subjects had 3 consecutive measurements in each eye with each device, which were conducted by two observers for the assessment of intraobserver repeatability and interobserver reproducibility. In the second session one week later, all subjects had an additional 3 consecutive scans by one observer for the assessment of intersession reproducibility. In addition, the mean values of the 3 measurements obtained by the first operator in the first session were used to assess the agreement and distribution between angle kappa measurements using the 3 devices [10]. All measurements were also taken under natural conditions between 10 am and 4 pm. Prior to taking examinations, all subjects were asked to blink with the purpose of optically smooth tear filming over the cornea. When considering dependent eyes for one individual, angle kappa in both right eyes and left eyes should be analysed.

### Statistical analysis

Statistical analysis was performed using SPSS software (version 23, SPSS Inc) and MedCalc software (version 14.12.02; MedCalc, Ostend, Belgium). Values are presented as the means ± standard deviations (SD). The Kolmogorov-Smirnov test was used to confirm the normality of all data distributions. A *P* value less than 0.05 was considered statistically significant.

To determine the intraoperator repeatability of each device, parameters such as the within subject standard deviation (Sw), test-retest repeatability, and intraclass correlation coefficient (ICC) were calculated for the 3 repeated measurements obtained by the first operator and the second operator [11]. The test-retest repeatability was defined as 2.77 Sw, which represents an interval within which 95% of the differences between measurements are expected to lie. The ICC is a reliability coefficient that evaluates the consistency for data sets of repeated measurements and is between 0 and 1 (ICC < 0.75: low reliability, 0.75 ≤ ICC ≤ 0.90: moderate reliability, and ICC > 0.9: high reliability).

Paired *t*-tests were used to assess the statistical significance of the difference between two means measured by any 2 of 3 devices. Pearson rank-order correlation coefficient values were calculated for the correlations by any 2 of 3 devices. The between-instrument agreement in estimating the mean preoperative angle kappa was analysed by the Bland-Altman method. The 95% limits of agreement (LoA) for each comparison (mean ± 1.96 SD) represented the range where 95% of all differences between two measurements were likely to fall.

A minimum of 34 patients was calculated using the formula recommended by Bland ^A^ when 3 repeated measurements were taken and a confidence interval of 20% was considered.

## Results

The study comprised 113 eyes of 113 patients: 60 eyes of 60 patients in right eyes and 53 eyes of 53 patients in left eyes. Table 1 shows the preoperative patient parameters. There was no statistically significant difference in age, axial length (AL), mean keratometry (Km), anterior chamber depth (ACD), nuclear opalescence (NO), and UDVA between right eyes and left eyes (all *P* > 0.05).

**Table 1:** Comparison of preoperative patient parameters in both eyes (Mean±SD)

Parameter	Right eyes	Left eyes	*t* Value	*P* Value
Age (y)	63.12±12.59	62.87±9.34	0.120	0.905
AL (mm)	24.36±2.49	24.32±2.29	0.094	0.925
Km (D)	44.02±2.23	44.07±1.45	-0.146	0.884
ACD (mm)	3.15±0.44	3.22±0.43	-0.771	0.442
NO (LOCS III)	1.89±0.60	2.01±0.53	-1.605	0.111
UDVA (logMAR)	0.48±0.12	0.50±0.12	-0.878	0.382

SD=standard deviations; AL=axial length; Km=mean keratometry; ACD=anterior chamber depth; NO=nuclear opalescence; LOCS III=lens opacities classification systems III; UDVA=uncorrected distance visual acuity; logMAR=logarithm of the minimum angle of resolution

### Intraoperator repeatability

Angle kappa measurements obtained by the 2 observers were highly repeatable for all 3 devices in both right eyes and left eyes (Table 2). The 2.77 Sw of both observers ranged from 0.03 to 0.08 mm. The ICC was higher than 0.96 in all cases.

**Table 2:** Intraobserver repeatability of angle kappa measurements by device for both eyes

Device	Mean±SD	Sw (mm)	2.77 Sw (mm)	ICC
Right eyes				
Pentacam				
1st	0.23±0.09	0.01	0.03	0.993
2nd	0.23±0.10	0.02	0.06	0.978
Lenstar				
1st	0.24±0.11	0.01	0.03	0.991
2nd	0.24±0.12	0.02	0.06	0.982
iTrace				
1st	0.31±0.15	0.02	0.06	0.978
2nd	0.32±0.16	0.02	0.06	0.980
Left eyes				
Pentacam				
1st	0.24±0.12	0.02	0.06	0.986
2nd	0.24±0.13	0.02	0.06	0.985
Lenstar				
1st	0.25±0.13	0.02	0.06	0.984
2nd	0.25±0.14	0.02	0.06	0.987
iTrace				
1st	0.34±0.11	0.02	0.06	0.985
2nd	0.35±0.13	0.03	0.08	0.964

SD=standard deviations; Sw=within-subject standard deviation; ICC=intraclass correlation coefficient

### Interoperator and intersession reproducibility

The interoperator reproducibility and intersession reproducibility of the angle kappa measurements were high with all 3 devices in both right eyes and left eyes (Table 3 and Table 4). The 2.77 Sw of interoperator reproducibility was 0.06 to 0.08 mm, and that of intersession reproducibility was 0.06 to 0.14 mm. The ICC was higher than 0.97.

**Table 3:** Intraobserver reproducibility of angle kappa measurements by device for both eyes

Device	Mean±SD	Sw (mm)	2.77 Sw (mm)	ICC
Right eyes				
Pentacam	0.23±0.10	0.02	0.06	0.990
Lenstar	0.24±0.11	0.02	0.06	0.982
iTrace	0.31±0.13	0.02	0.06	0.984
Left eyes				
Pentacam	0.24±0.12	0.02	0.06	0.987
Lenstar	0.25±0.14	0.02	0.06	0.983
iTrace	0.34±0.15	0.03	0.08	0.973

SD=standard deviations; Sw=within-subject standard deviation; ICC=intraclass correlation coefficient

**Table 4:** Intersession reproducibility of angle kappa measurements by device for both eyes

Device	Mean±SD	Sw (mm)	2.77 Sw (mm)	ICC
Right eyes				
Pentacam	0.23±0.12	0.02	0.06	0.991
Lenstar	0.24±0.12	0.03	0.08	0.988
iTrace	0.31±0.15	0.04	0.11	0.977
Left eyes				
Pentacam	0.24±0.13	0.03	0.08	0.987
Lenstar	0.25±0.15	0.04	0.11	0.983
iTrace	0.34±0.16	0.05	0.14	0.971

SD=standard deviations; Sw=within-subject standard deviation; ICC=intraclass correlation coefficient

### The difference, correlation, and agreement of angle kappa

Table 5 shows the difference, correlation, and agreement by any 2 of 3 devices in both right eyes and left eyes. There were no statistically significant differences in the angle kappa, between Pentacam and Lenstar (right eyes: *P*=0.354; left eyes: *P*=0.181), while there were statistically significant differences in the angle kappa between Pentacam and iTrace and between Lenstar and iTrace (*P<*0.05). Similarly, a strong correlation was found for the angle kappa between Pentacam and Lenstar (*r*=0.907 to 0.918), while a weak or moderate correlation was found for the angle kappa between Pentacam and iTrace and between Lenstar and iTrace (*r*=0.292 to 0.618). Figures 3 and 4 show the interdevice agreement for mean angle kappa. The 95% LoA between Pentacam and Lenstar was narrower (right eyes: -0.09 to 0.08 mm; left eyes: -0.11 to 0.09 mm), while the 95% LoA was wider in the comparisons involving the iTrace.

**Table 5:** Comparison of angle kappa by any 2 of 3 devices in both eyes

Device Pairing		Right eyes	Left eyes
Pentacam-Lenstar	*P* Value	0.354	0.181
	*r* Value	0.907	0.918
	95% LoA(mm)	-0.09, 0.08	-0.11, 0.09
Pentacam-iTrace	*P* Value	<0.001	<0.001
	*r* Value	0.294	0.598
	95% LoA(mm)	-0.37, 0.21	-0.30, 0.10
Lenstar-iTrace	*P* Value	<0.001	<0.001
	*r* Value	0.292	0.618
	95% LoA(mm)	-0.38, 0.22	-0.30, 0.12

LoA= limits of agreement

### Comparison of pupil diameter

The pupil diameters measured by Pentacam, Lenstar and iTrace in the right eyes were (3.51± 0.54) mm, (3.56±0.51) mm, and (3.71±0.46) mm, respectively, and those in the left eyes were (3.60±0.50) mm, (3.69±0.53) mm, and (3.80±0.38) mm, respectively. There were no statistically significant differences in the pupil diameter between Pentacam and Lenstar or between Lenstar and iTrace in either eye (all *P* > 0.05). However, there were statistically significant differences in the pupil diameter with Pentacam and iTrace in both right eyes and left eyes (*P*= 0.02 and 0.011). In addition, no significant differences in the pupil diameter were noted between the right eyes and left eyes by Pentacam, Lenstar and iTrace (*P*= 0.325, 0.207, and 0.305).

### The distribution and percentage of the preoperative angle kappa

**Fig. 2:**
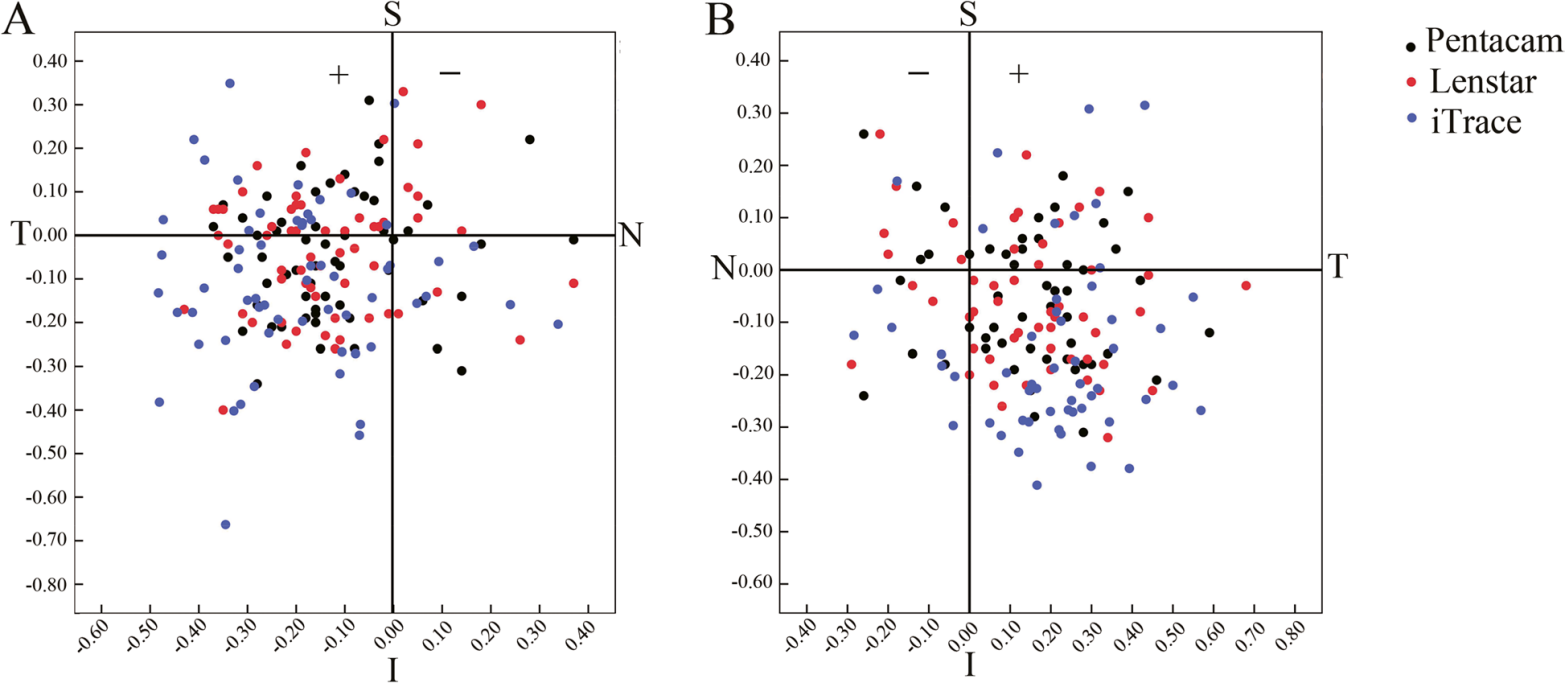
The distribution of angle kappa in right (A) and left (B) eyes. The **+** and **–** sign represent the positive and negative angle kappa, respectively (S= superior; I= inferior; T= temporal; N= nasal).

Table 6 shows the distribution and percentage of the preoperative angle kappa. The distribution of positive angle kappa by Pentacam, Lenstar and iTrace in right eyes was 50 eyes (83.33%), 49 eyes (81.67%), and 53 eyes (88.33%), respectively, and in left eyes was 42 eyes (79.25%), 42 eyes (79.25%), and 45 eyes (84.91%), respectively (Figure 2). There were no statistically significant differences in the percentage of positive angle kappa with any 2 of 3 devices in right eyes and left eyes (*P* > 0.05).

**Table 6:** The distribution and percentage of angle kappa in 8 positions in both eyes [N (%)]

Parameter	Right eyes			Left eyes		
	Pentacam	Lenstar	iTrace	Pentacam	Lenstar	iTrace
Superior nasal	3(5.00)	7(11.67)	0(0)	5(9.43)	6(11.32)	1(1.89)
Inferior nasal	6(10.00)	4(6.67)	6(10.00)	4(7.55)	3(5.66)	7(13.21)
Superior temporal	19(31.67)	21(35.00)	16(26.67)	13(24.53)	10(18.87)	7(13.21)
Inferior temporal	29(48.33)	26(43.33)	37(61.67)	28(52.83)	31(58.49)	37(69.81)
Nasal	0(0)	0(0)	0(0)	0(0)	0(0)	0(0)
Temporal	2(3.33)	2(3.33)	0(0)	1(1.89)	1(1.89)	1(1.89)
Superior	0(0)	0(0)	1(1.67)	1(1.89)	0(0)	0(0)
Inferior	1(1.67)	0(0)	0(0)	1(1.89)	2(3.77)	0(0)

SD= standard deviations; N=the number of eyes; %=percentage of data expressed as eyes

**Fig. 3:**
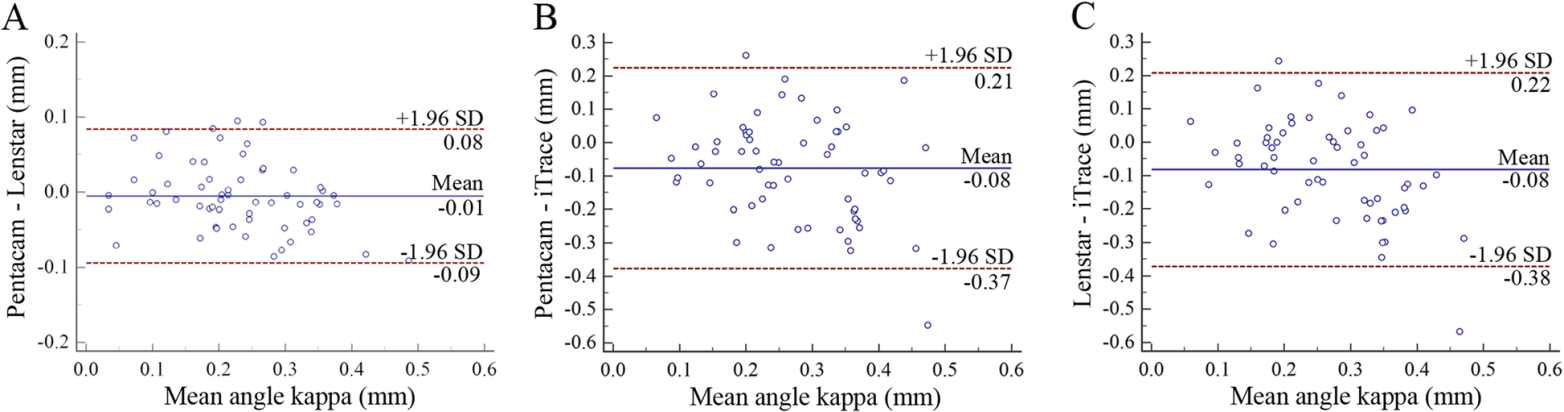
Bland–Altman plot of between Pentacam and Lenstar (A), between Pentacam and iTrace (B), and between Lenstar and iTrace (C) for angle kappa in right eyes

**Fig. 4:**
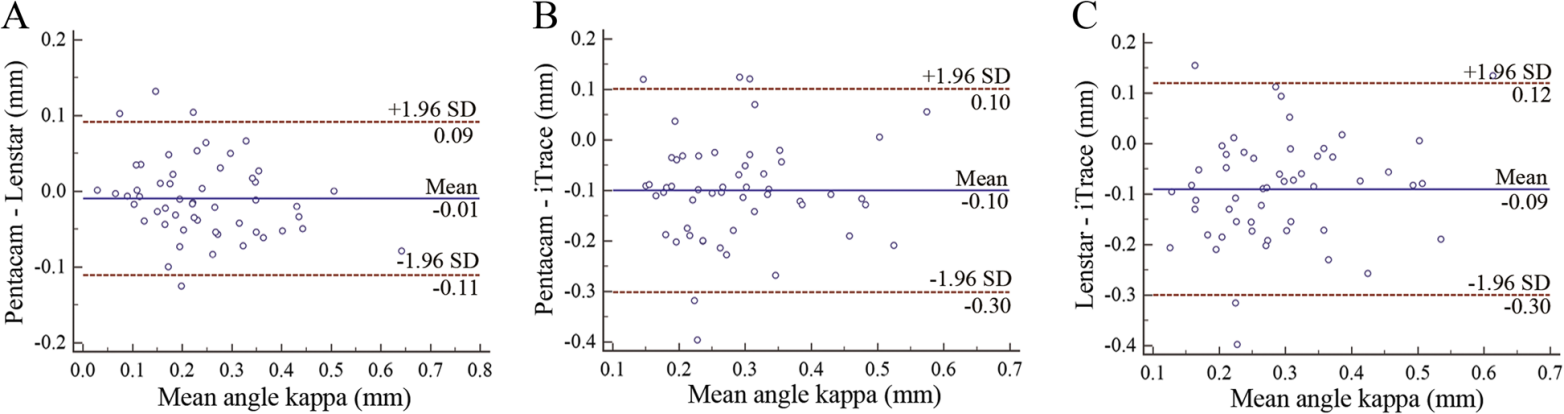
Bland–Altman plot of between Pentacam and Lenstar (A), between Pentacam and iTrace (B), and between Lenstar and iTrace (C) for angle kappa in left eyes

### Comparison of angle kappa in both right eyes and left eyes

The left eyes were found to give higher average angle kappa values than the right eyes, and there were no significant differences between the right eyes and left eyes in angle kappa by Pentacam, Lenstar and iTrace (Pentacam, Lenstar and iTrace: *P*=0.587, 0.505, and 0.252, respectively).

## Discussion

The human eye is not a perfect optical system. The larger the discrepancy between the visual axis and pupillary axis or visual axis and optical axis is, the lower the visual quality. In the MIOL of eyes with a large angle kappa, the light might pass the center of the macula through other diffractive rings instead of the central area of the MIOL, which is also known as functional IOL decentration [12], thereby leading to photic phenomena and surgical aberrations [13, 14]. Therefore, according to the angle kappa, adjusting the position of the MIOL may help reduce photic phenomena [13, 15]. In addition, some studies also indicated that more than 0.5 mm of preoperative angle kappa should be carefully considered in cataract patients implanted with diffractive or refractive types of MIOL [6, 13, 16]. A previous study indicated that the size of angle kappa was calculated using the chord length of X and Y, and the value of angle kappa played an important role [6]. For patients with a larger angle kappa, the choice to implant an MIOL should be carefully evaluated. When the angle kappa was greater than 0.4 mm, the incidence of glare and halo increased and when it was greater than 0.5 mm, patients’ visual quality decreased. Hence, the measurement of angle kappa is particularly important, and this received increasing attention from ophthalmologists. To our knowledge, ours is the first study to comprehensively assess the intraobserver repeatability, interobserver reproducibility, and intersession reproducibility of angle kappa measurements using 3 instruments (Pentacam, Lenstar, iTrace). In this study, these devices provided highly precise angle kappa measurements between the 3 devices. The 3 devices are noncontact optical methods, which decrease risks for corneal abrasion or infection, and increase patient comfort and acceptability. However, when the refractive medium is opaque or the tear film is unstable, these devices cannot successfully obtain a measurement.

A previous study showed that the angle kappa value was different between the different instruments [15]. A study found a strong correlation between synoptophore and Orbscan II measurements, but angle kappa obtained from Orbscan II was dramatically higher than that obtained from synoptophore [17]. Another study showed that Orbscan II measured significantly higher angle kappa than Galilei G4. However, angle kappa did not change significantly for different accommodation levels [15]. In addition, a report designed a new method using UBM and corneal topography to calculate the angle kappa and compared the new method with Orbscan II, which found good correlations and agreement between the two methods. However, there was a statistically significant difference in angle kappa measured by the devices [2]. One explanation for the discrepancy between measurements might be the different principles in different devices, producing slightly different results. The agreement between devices could be significant in research reports that use angle kappa as an important parameter before surgery. In our study, different from the angle displaying angle kappa, this study represented the angle kappa through X-Y Cartesian coordinates to obtain the distribution of angle kappa and compared the chord length of X and Y. In addition, angle kappa was affected by dependent eyes and pupil diameter for one individual [18, 19]. Angle kappa in both right eyes and left eyes should be analysed separately, and there were no significant differences in pupil diameter between Pentacam and Lenstar in our study. This excluded the effect dependent eyes and pupil diameter on angle kappa, which showed that our results were more convincing.

Our study demonstrated that angle kappa for a majority of cataract patients was positive (nasal light reflex) with 3 different devices, which was consistent with those obtained by other research [4]. This might be related to the anatomical location of the macular fovea on the temporal side of the intersection of the pupil axis at the posterior pole of the eyeball. In addition, we found no statistically significant differences in the percentage of positive angle kappa between any 2 of the 3 types. Thus, 3 instruments may replace each other in the measurement of positive angle kappa. Moreover, there were no differences, strong correlations, and good agreement in the angle kappa between Pentacam and Lenstar for cataract patients in either eye. Therefore, the measurement of angle kappa obtained from Pentacam and Lenstar can be trusted and referred by each other in clinical practice, especially for patients with large angle kappa or poor coordination in clinical practice. However, the angle kappa measured by iTrace was significantly different when compared with Pentacam or Lenstar, which may be because the 3 devices have different principles. In addition, iTrace needed automatic radiation 3 times to obtain a result while Pentcam and Lenstar only identified automatically 1 time. Compared with Pentcam and Lenstar, iTrace had a greater impact on the stability of the tear film, thereby affecting the measurement of angle kappa. Hence, iTrace should be carefully considered for the measurement of angle kappa in dry eyes. In clinical practice, due to the lower wavelength compared to Lenstar, iTrace decreased issue penetration. In the case of mature cortex cataracts, grade IV hard nucleus cataracts, and high myopia, the laser cannot enter the eye, leading to the inability to measure the angle kappa. Hence, iTrace should be carefully considered for the measurement of angle kappa in mature cortex cataracts, grade IV hard nucleus cataracts, and high myopia.

This study also found that there was a slight tendency towards higher angle kappa values in left eyes when compared to right eyes and there were no significant differences between right eyes and left eyes in angle kappa. The underlying cause is likely eye dominance, mean anatomic difference of lens and corneal radii head posture, facial asymmetries, and eye habit, but these do not clearly indicate which factor is the main reason for the difference between left eyes and right eyes. Therefore, it should be carefully considered for patients with left eyes in strabismus surgery, corneal refractive surgery, and implantation of MIOLs. In addition, angle kappa decreases significantly with AL and age [20, 21], but there was no significant difference between sexes. Furthermore, some other studies have shown that the angle kappa is affected by a variety of factors, such as different postures [22], changes in illumination [23, 24], strabismus [25], and refraction errors (myopia, emmetropia, and hypermetropia) [17]. Therefore, clinicians must consider comprehensive factors affecting the angle kappa account when performing surgery on angle kappa in clinical work.

This study has several limitations. First, there was a relatively small number of patients in this research. Therefore, it is necessary to study a larger sample population about angle kappa in the future to provide further guidance for refractive cataract surgery. Second, there was no control group (normal eyes), so angle kappa in the normal eyes measured by three instruments was not compared. In addition, whether there will be good agreement in the normal eyes between Pentacam and Lenster can be confirmed. Third, Pentacam and Lenstar represent angle kappa to display X–Y Cartesian coordinates between the corneal vertex and pupil center, while iTrace displays X–Y Cartesian coordinates between the visual axis and pupil center. From the principle of measurement, angle kappa by iTrace is closer to the definition of angle kappa. Therefore, iTrace is the most correct way to measure the angle kappa, but it is more susceptible to poor fixation. Fourth, the latest research has shown that angle alpha may be an alternative or even better predictor for MIOL suitability. A similar discovery has been completed by our research group, and related articles are being submitted.

## Conclusions

Our results suggest that the 3 devices provided high intraoperator repeatability and interoperator and intersession reproducibility for angle kappa measurements, and the measurement of preoperative angle kappa in the eyes of patients with cataracts by Pentacam and Lenstar has good agreement.

## Data Availability

The datasets used during the current study are available from the corresponding author on reasonable request.

## Abbreviations

IOL: intraocular lens
MIOLs: multifocal intraocular lens
SD: standard deviation
LOA: limit of agreement
UDVA: uncorrected distance visual acuity
LoA: limits of agreement
AL: axial length; Km: mean keratometry
ACD: anterior chamber depth
NO: nuclear opalescence
LOCS III: lens opacities classification systems III
logMAR: logarithm of the minimum angle of resolution
